# Corrosion Behavior of Cast Iron in Freely Aerated Stagnant Arabian Gulf Seawater

**DOI:** 10.3390/ma8052127

**Published:** 2015-04-27

**Authors:** El-Sayed M. Sherif, Hany S. Abdo, Abdulhakim A. Almajid

**Affiliations:** 1 Deanship of Scientific Research (DSR), Advanced Manufacturing Institute (AMI), King Saud University, P.O. Box 800, Riyadh 11421, Saudi Arabia; E-Mails: enghany2000@yahoo.com (H.S.A.); aalmajid@ksu.edu.sa (A.A.A.); 2 Electrochemistry and Corrosion Laboratory, Department of Physical Chemistry, National Research Centre (NRC), Dokki, Cairo 12622, Egypt; 3 Mechanical Design and Materials Department, Faculty of Energy Engineering, Aswan University, Aswan 81521, Egypt; 4 Department of Mechanical Engineering, College of Engineering, King Saud University, P.O. Box 800, Riyadh 11421, Saudi Arabia

**Keywords:** Arabian Gulf seawater, cast iron, corrosion, electrochemical measurements, weight-loss

## Abstract

In this work, the results obtained from studying the corrosion of cast iron in freely aerated stagnant Arabian Gulf seawater (AGS) at room temperature were reported. The study was carried out using weight-loss (WL), cyclic potentiodynamic polarization (CPP), open-circuit potential (OCP), and electrochemical impedance spectroscopy (EIS) measurements and complemented by scanning electron microscopy (SEM) and energy dispersive X-ray (EDX) investigations. WL experiments between two and 10 days’ immersion in the test electrolyte indicated that the weight-loss the cast iron increases with increasing the time of immersion. CPP measurements after 1 h and 24 h exposure period showed that the increase of time decreases the corrosion via decreasing the anodic and cathodic currents, as well as decreasing the corrosion current and corrosion rate and increasing the polarization resistance of the cast iron. EIS data confirmed the ones obtained by WL and CPP that the increase of immersion time decreases the corrosion of cast iron by increasing its polarization resistance.

## 1. Introduction

Cast iron is a durable and fire-resistant material that is used in the home and industry. It is a complex material with stable and meta-stable phases and has elements in the solution, which influence the extent and stability of the desirable properties not obtained by other alloys. Cast iron is primarily an alloy of iron that contains carbon content of 2%–5% and at least 1% silicon, in addition to traces of manganese, sulfur, and phosphorus. Cast iron has a wide variety of properties such as cast complex shape with low cost, low melting temperature, high fluidity when molten, it does not form undesirable surface film when poured due to less reactivity with molten materials and has slight to moderate shrinkage during solidification and cooling [1]. It is also hard, brittle, nonmalleable, and more fusible than steel. Cast irons are widely used materials for components handling seawater and brine such as large intake, recycling and blow down pumps for desalination and power plants, and other hydraulic machinery. In particular, diesel engine cylinder liners are manufactured almost exclusively from flake graphite grey irons [2,3,4,5].

Upon manufacture, cast iron develops a protective film or scale on the surface which makes it initially more resistant to corrosion than wrought iron or mild steel [6]. It has been reported [7] that alloying elements play an important role in the susceptibility of cast irons to corrosion attack. Where, its corrosion depends mainly on the percent of silicon existed in the alloy; the higher the silicon content, the higher the corrosion resistance [7]. The corrosion of cast iron in different environments has attracted few investigators. Yilbas *et al.* [8] have reported the improved corrosion resistance of cast iron surface treated with laser gas assisted of dual matrix structured. Al-Hashem *et al.* [9] studied the effect of microstructure of nodular cast iron on its cavitation corrosion behavior in seawater and found that the surface becomes very rough with large size cavity pits. Olawale *et al.* [10] have evaluated the corrosion behavior of grey cast iron (GCI) and low alloy steel (LAS) in cocoa liquor and well water and found that LAS has better corrosion resistance than GCI in both media, and cocoa liquor is more aggressive than well water. They [10] also recommended that LAS is a better material for piping and pumping systems in cocoa processing industries than GCI.

The present work aims at investigating the corrosion behavior of cast iron after different immersion periods of time in the naturally aerated stagnant AGS solution at room temperature using gravimetric weight-loss, varied electrochemical, and spectroscopic techniques. The weight-loss method was employed to report the dissolution behavior for the cast iron over 10 days’ immersion. The electrochemical techniques such as CPP, EIS, and OCP and were for reporting the electrochemical and kinetic parameters for the cast iron under investigation after its immersion for 1 h and 24 h in AGS medium. Spectroscopic investigations using SEM and EDX profile analysis were to understand the formed corrosion products onto the surface of cast iron that was immersed for 10 days’ immersion in AGS.

## 2. Results and Discussion

### 2.1. Weight-Loss Measurements and SEM/EDX Investigations

**Figure 1:**
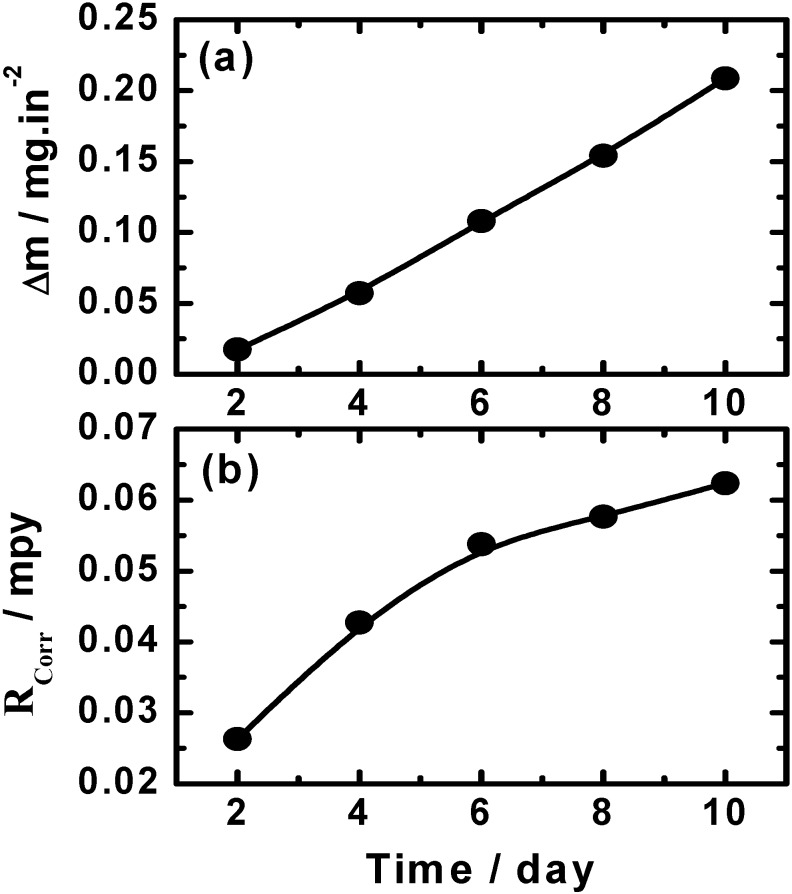
Change of (**a**) the dissolution rate (Δm, mg/in^2^) and (**b**) the corrosion rate (*R*_Corr_, mpy) with time for the cast iron coupons in the Arabian Gulf seawater (AGS) solutions.

The variation of (a) the weight-loss (Δm, mg/in^2^) and (b) the corrosion rate (*R*_Corr,_ mpy) *vs.* time for the cast iron coupons in 300 cm^3^ of aerated stagnant solutions of AGS are shown in Figure 1. The values of Δm and *R*_Corr_ over the exposure time were calculated as reported in our previous work as following [11]:

(1) $$\Delta m\;\;=\frac{m_{1}\;-\;m_{2}}{A}$$

(2) $$R_{\text{Corr}}\;\;=\frac{543\;\Delta m}{Dt}$$

where, m_1_ and m_2_ are the weighs of the cast iron coupon per mg before and after its immersion in the test solution, *A* is the area of the cast iron coupon in inch^2^, *D* is the density of cast iron (*D* = 7.563 g/cm^3^), and *t* is the exposure time (h).

One can see from Figure 1a that the values of Δm increased with time due to the aggressiveness attack of the corrosive ions present in the seawater toward the cast iron surface. It is well known that the cathodic reaction for metals and alloys in near neutral solutions is the oxygen reduction according to the following equation:

2H_2_O + O_2_ + 4e^−^ = 4OH^−^ (3)

On the other hand, the anodic reaction of iron in aerated neutral solutions is the dissolution of metallic iron (Fe°) to ferrous cations (Fe^2+^) as follows,

Fe = Fe^2+^ + 2e^−^ (4)

The dissolution of iron gets activated due to the consumption of the produced electrons via the cathodic reaction at the anodic one, which could lead to the increased weight-loss with time, Figure 1a. Similar to the increase of Δm with time, the values of K_Corr_ also increased with increasing the immersion time, particularly at short periods, as can been seen from Figure 1b. This increment slightly decreased at longer immersion time due to the formation of thick corrosion products that decreases the attack of the corrosive species present in the seawater and thus decreases the corrosion rate.

**Figure 2:**
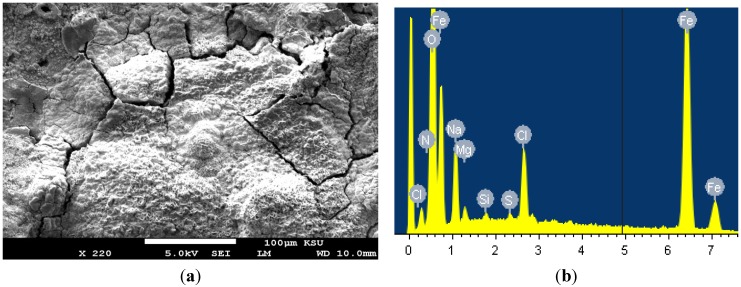
(**a**) SEM micrograph obtained for the cast iron coupons after its immersion in AGS for 10 days; and (**b**) the EDX profile analysis obtained for the surface shown in the SEM image.

In order to understand the morphology and the composition of the corrosion products formed on the cast iron coupon after its immersion in the seawater for 10 days, SEM/EDX investigations for the surface of the coupon were carried out. Figure 2a shows the SEM image obtained for the cast iron surface at different magnifications after 10 days’ immersion in the aerated stagnant AGS solution, and Figure 2b represents the EDX profile analysis for the area of the surface shown in Figure 2a. It is clearly seen from the SEM image that the surface of the cast iron is fully covered with thick layers of corrosion products due to the immersion of the coupon in AGS for long time, 10 days. The atomic percentages of the components found on the surface were 56.87% O, 32.57% Fe, 9.64% Na, 3.50% Cl, 1.04% Mg, 0.29% Si, and 0.27% S. The very high content of oxygen and iron indicates that the layers formed on the cast iron surface after 10 days immersion in AGS were most probably iron oxide films. This can be explained on the light of the overall reaction that occurs on the iron surface as follows,

Fe + ½ O_2_ + H_2_O → Fe(OH)_2_ (5)

This ferrous hydroxide formed layer reacts, further in the presence of excess oxygen in the solution, to building up the final corrosion product Fe_3_O_4_ (magnetite) according to the following reaction [12];

3Fe(OH)_2_ + ½ O_2_ → Fe_3_O_4_ + 3H_2_O (6)

According to Mohebbi and Li in a similar study [13], both Fe^3+^ and Fe^2+^ ion species existed in the corrosion product, which indicates that the electrons generated by oxidation of iron (cathodic reaction) can be readily consumed by the oxygen present in the electrolyte solution through the porous conductive corrosion layer. The presence of Cl ions gives indications on the increase of weight-loss and corrosion rate of cast iron with time due to possible reactions of Cl^−^ with the inner surface of iron [13]. This is due to the dissolution of iron as shown in Equation (4), which would lead to the formation of FeCl_2_ and FeC1_3_ in the solution. The concentration of FeCl_2_ and FeC1_3_ at this condition is at saturation and would precipitate to form a porous mixed film(s) of FeCl_2_ and FeC1_3_ on the iron electrode surface [14],

Fe + 2Cl^−^ = FeCl_2_ + 2e^−^ (7)

FeCl_2_ + Cl^−^ = FeCl_3_ + e^−^ (8)

In addition to the iron oxides, the presence of Mg, Na and Si indicates that their oxides might have also formed on the surface.

### 2.2. Cyclic Potentiodynamic Polarization (CPP) Measurements

**Figure 3:**
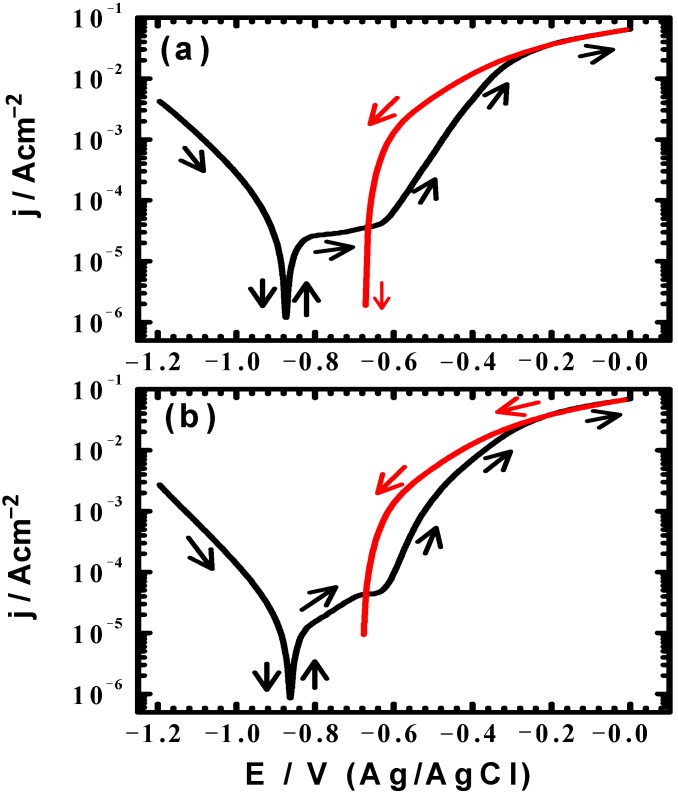
Cyclic potentiodynamic polarization curves obtained for the cast iron electrode after its immersion in AGS solutions for (**a**) 1 h and (**b**) 24 h, respectively.

Cyclic potentiodynamic polarization testing were conducted to measure the corrosion parameters such as cathodic (*β*_c_) and anodic (*β*_a_) Tafel slopes, corrosion potential (*E*_Corr_), pitting potential (*E*_Pit_), protection potential (*E*_Prot_), corrosion current (*j*_Corr_), polarization resistance (*R*_P_) and corrosion rate (*R*_Corr_). Figure 3 shows the CPP curves obtained for the cast iron electrode after its immersion in AGS solutions for (a) 1 h and (b) 24 h, respectively. The corrosion parameters obtained from the CPP curves shown in Figure 3 are list in Table 1. The values of *β*_c_ and *β*_a_ were determined after at least 50 mV away from E_Corr_ and at least one decade of current densities (*j*_Corr_). The values of *E*_Corr_ and *j*_Corr_ were obtained from the intersection of the extrapolation of anodic and cathodic Tafel lines located next to the linearized current regions. The values of polarization resistance, *R*p, and corrosion rate, *R*_Corr_, for the cast iron were calculated according to the following equations [12,15] corrosion as follows:

(9) $$R_{P}=\frac{1}{j_{\text{Corr}}}\left(\frac{\beta_{c}.\beta_{a}}{2.3\;\left(\beta_{c}+\;\beta_{a}\right)}\right)$$

(10) $$R_{\text{Corr}}\;=\frac{j_{\text{Corr}}\;k\;\;\;\;\;E_{W}\;}{d\;\;A}$$

Where, k is a constant that defines the units for the corrosion rate (=3272 mm amp^1^cm^−1^ y^−1^); E_W_ the equivalent weight in grams/equivalent of cast iron alloy (E_W_ = 27.9 grams/equivalent); d the density in gcm^−3^ (=7.563); and A the area of the exposed surface of the electrode in cm^2^.

It is clearly seen from Figure 3 that the anodic branch for the cast iron shows a passive region whether the measurement was taken after 1 h (Figure 3a) or after 24 h (Figure 3b). This passive region was formed due to the formation of an oxide film as depicted by Equations (5) and (6). Where, the formed ferrous hydroxide reacted with more oxygen to form the top layer of magnetite corrosion product, Fe_3_O_4_. The current then increased rapidly in the anodic side due to the breakdown of the formed oxide film and the occurrence of pitting corrosion. This was indicated by the higher current values in the backward direction of the scanned potential and the appearance of a hysteresis loop, the area of which decreased by increasing immersion time from 1h to 24 h. It is well known that AGS contains corrosive species such as chloride ions, which attacks the weak and flowed areas of the oxide film formed on the iron surface in the passive region causing its breakdown and corrosion via pitting by chloride ions attack as depicted in Equations (7) and (8).

**Table 1:** Polarization parameters obtained for the cast iron electrode after its immersion for 1 h and 24 h in the Arabian Gulf seawater (AGS).

Immersion time	Polarization Parameters							
	*β*_c_/ mVdec^−1^	*E*_Corr_/ mV	*β*_a_/ mV/dec^−1^	*j*_Corr_/ µA cm^−2^	*E*_Pit_/ mV	*E*_Prot_/ mV	*R*_p_/ Ω cm^2^	*R*_Corr_/ mmpy
AGS (1 h)	95	−860	230	20	−630	−665	1471	0.2414
AGS (24 h)	105	−825	210	11	−620	−680	2767	0.1328

The parameters shown in Table 1 show that the increase of immersion time from 1h to 24 h shifted *E*_Corr_ and *E*_Pit_ to the less negative values, while *E*_Prot_ was shifted to the more negative values. This indicates that both general and pitting corrosion decreased with increasing the time of immersion for the cast iron in AGS. Table 1 also shows that the increase of time before measurements decreased the values of j_Corr_ and *R*_Corr_ as well as increased the polarization resistance (*R*p).

### 2.3. Open-Circuit Potential (OCP) and Electrochemical Impedance Spectroscopy (EIS) Measurements

**Figure 4:**
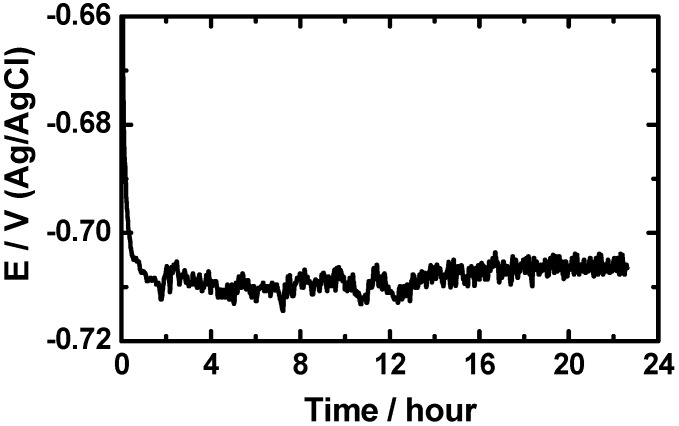
Change of the open-circuit potential with time for the cast iron electrode in the AGS solution.

Figure 4 shows the change of the OCP with time for the cast iron electrode in the AGS solution. It is seen from Figure 4 that the initial potential of iron rapidly increased towards the more negative values in the first few minutes due to the dissolution of a preformed air oxide film. The potential then slightly shifted in the more negative direction with the appearance of some fluctuations by increasing time for the first 12 h. This more negative shift might have resulted from the dissolution of iron by the corrosive ions attack such as chlorides. Finally, the potential very slightly decreases again towards the less negative direction till the end of the experiment as a result of the oxide and/or corrosion product layers on the surface.

**Figure 5:**
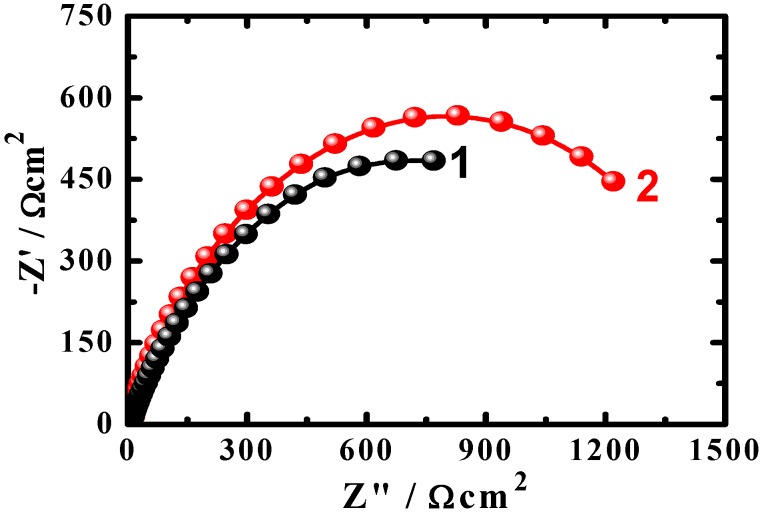
Typical Nyquist obtained for the cast iron electrode after (**1**) 1 h and (**2**) 24 h immersion in the AGS solution, respectively.

**Figure 6:**
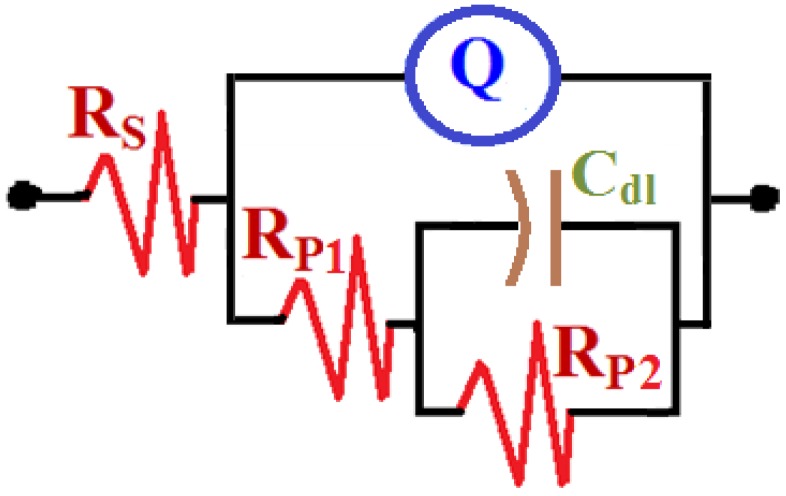
The equivalent circuit model used to the fit the EIS data shown in Figure 5; the symbols of the equivalent circuit are defined in the text and the values are listed in Table 2.

EIS method is a powerful technique that has been used in studying corrosion and corrosion inhibition of metals and alloys in various corrosive media [14,15,16,17,18,19,20,21]. Typical Nyquist obtained for the cast iron electrode after (1) 1 h and (2) 24 h immersion in the AGS solution, respectively, are shown in Figure 5. The spectra represented in Figure 5 were analyzed by best fitting to the equivalent circuit model depicted in Figure 6. The EIS parameters obtained by fitting this circuit are listed in Table 2 and can be defined according to the usual convention as follows; R_S_ represents the solution resistance, Q is the constant phase elements (CPEs), Cdl is the double layer capacitance, Rp_1_ is the polarization resistance for the solution/cast iron interface and may be defined as the charge transfer resistance of the cathodic reduction reaction of the cast iron, and Rp_2_ is another polarization resistance for the corrosion product/cast iron interface [22].

**Table 2:** Parameters obtained by fitting the EIS data shown in Figure 5 with the equivalent circuit shown in Figure 6 for the electrode after its immersion for 1 h and 24 h in AGS.

Immersion Time	Kinetic EIS Parameters					
	*R*_S_/ Ω cm^2^	*Q*		*R*_P1_/ Ω cm^2^	*C*dl/ F cm^−^^2^	*R*_P2_/ Ω cm^2^
		*Y*_Q_/F cm^−^^2^	*n*			
AGS (1 h)	5.814	0.000948	0.72	0.3977	5.232 × 10^−^^5^	1047
AGS (24 h)	6.127	0.000539	0.72	40.36	2.374 × 10^−^^5^	1571

It is seen from Figure 5 that the cast iron showed only one semicircle whether the immersion time for the cast iron before measurement was 1 h or 24 h. The diameter of the obtained semicircle got wider by increasing the immersion time to 24 h, which indicates that the resistance against corrosion increased by increasing the exposure period of time from 1h to 24 h. The values of *R*_S_, *R*p_1_ and *R*p_2_ that are listed in Table 2 recorded higher values for the cast iron immersed in AGS for 24 h compared to those obtained after only 1 h. This is due to the increase of the corrosion resistance for the surface of the cast iron with increasing time. The constant phase elements (*Q*, CPEs) with its n values exactly 0.72 for the cast iron after 1 h and 24 h immersion in the AGS electrolyte represent double layer capacitors with some pores that allow the dissolution of iron [14,15,16,17], which agrees with the work of Mohebbi and Li [13]. Where and depending on the value of *n*, a CPE can represent resistance (*Z*(CPE) = *R*, *n* = 0), capacitance (*Z*(CPE) = Cdl, *n* = 1) or Warburg impedance for (*n* = 0.5). Therefore, the CPE for iron and steel is substituted for the capacitor to fit the semicircle more accurately. According to Zhang *et al.*, the admittance and the impedance of a CPE at this condition can be defined by the following equations, respectively [16].

*Y*_CPE_ = *Y*_0_ (jω)^n^ (11)

*Z*_CPE_ = (1/*Y*_0_) (jω)^−^^n^ (12)

where, *Y*_0_ is the modulus; ω is the angular frequency; and n is the phase. The decrease of the CPEs and *C*dl values byincreasing the immersion period to 24 h reveals also that elongating time lowers the corrosion of the cast iron [16,23].

**Figure 7:**
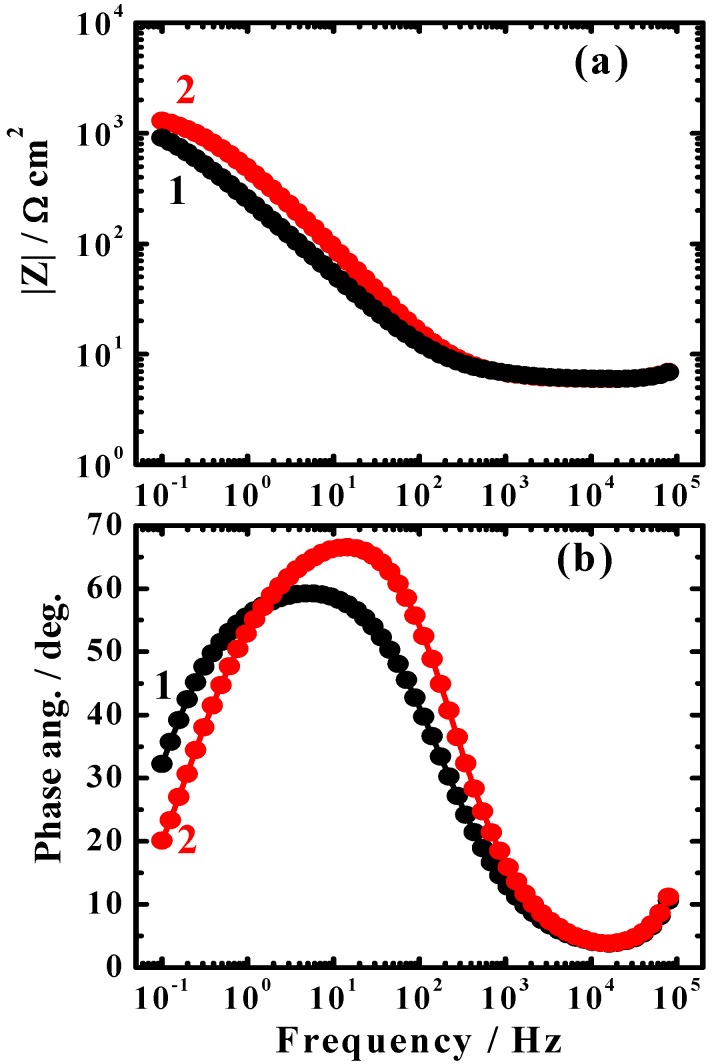
Typical Bode (**a**) impedance of the interface, |*Z*|; and (**b**) phase angle plots obtained for the cast iron electrode after (**1**) 1 h and (**2**) 24 h immersion in the AGS solution, respectively.

Figure 7 shows the typical Bode (Figure 7a) impedance of the interface, |*Z*|, and (Figure 7b) phase angle plots obtained for the cast iron electrode after (1) 1 h and (2) 24 h immersion in the AGS solution, respectively. It is obvious that the increase of immersion time to 24 h before measurements increased the impedance |*Z*| values (Figure 7a). It has been reported by Mansfeld *et al.* [24] that the surface is more protected when higher |*Z*| values are shown, particularly at the low frequency region. The increase of immersion time also increased the maximum degree of the phase angle as can be seen from Figure 7b. This confirms that the better corrosion resistance of the cast iron with increasing time. The EIS Nyquist and Bode plots were consistent with each other and both are in good agreement with the data obtained by weight-loss and cyclic polarization measurements.

## 3. Experimental Section

Cast iron with the chemical compositions (in wt %) listed in Table 3 was employed in the present study. The Arabian Gulf seawater (AGS) was brought directly from the Arabian Gulf at the eastern region of Saudi Arabia. For electrochemical measurements, a conventional electrochemical cell accommodates only 200 cm^3^ and a three-electrode configuration was used. The three electrodes were the cast iron, platinum foil, and an Ag/AgCl electrode (in 3.0 M KCl), which were used as working, counter, and reference electrodes, respectively. The cast iron electrode had a square shape with dimensions of 1 cm × 1 cm × 3 cm; the exposed surface area of the electrode to the electrolytic AGS solution was only 1 cm^2^. Accordingly, the working electrode was prepared by welding an insulated copper wire to one face of the cast iron electrode and cold mounted in resin. The surface of the cast iron electrode to be exposed to the solution was first ground successively with metallographic emery paper of increasing fineness of up to 600 grits and further with 5 μm, 1 μm, 0.5 μm, and 0.3 μm alumina slurries (Buehler). The electrode was then washed with doubly distilled water, degreased with acetone, washed using doubly distilled water again and finally dried with tissue paper.

**Table 3:** Chemical compositions of the cast iron that has be employed in the present study.

Element	C	Si	Mn	P	Mg	Ti	W	Cr	Cu	Zn	S	Ce	Fe
Wt %	4.58	2.13	0.27	0.08	0.07	0.04	0.03	0.02	0.02	0.02	0.02	0.01	Balance

The weight loss experiments were carried out using rectangular cast iron coupons that had the same chemical composition as cast iron electrodes. The coupons had a dimension of 4.0 cm length, 2.0 cm width, and 0.4 cm thickness and the exposed total area of 54 cm^2^. The coupons were polished and dried as for the case of cast iron electrodes, weighed, and then suspended in 300 cm^3^ solutions of AGS for different exposure periods (2–10 days).

The SEM investigation and EDX analysis were obtained on the surface of a cast iron samples after its immersion in open to air stagnant AGS solution for 10 days. The SEM/EDX analysis was collected on cast iron samples with dimensions of 1 cm × 1 cm × 0.4 cm that were cut from the coupons used in the weight-loss test. The SEM images were carried out by using a JEOL model JSM-6610LV (Japanese made) scanning electron microscope with an energy dispersive X-ray analyzer attached.

An Autolab Potentiostat-Galvanostat (Metrohm Autolab B.V., Amsterdam, The Netherlands, PGSTAT20 computer controlled) operated by the general purpose electrochemical software (GPES) version 4.9 (Metrohm, Amsterdam, The Netherlands) was used to perform the electrochemical experiments. Cyclic potentiodynamic polarization (CPP) curves were obtained by scanning the potential in the forward direction from −1.20 V to 0.0 V *vs.* Ag/AgCl at a scan rate of 0.001 V/s. The potential was scanned again in the backward direction at the scan rate till the end of the run. The open-circuit potential measurements were carried out for 24 h at room temperature. The electrochemical impedance spectroscopy (EIS) tests were performed at corrosion potentials over a frequency range of 100 kHz–100 mHz, with an ac wave of ±5 mV peak-to-peak overlaid on a dc bias potential, and the impedance data were collected using Powersine software at a rate of 10 points per decade change in frequency. Each experiment was carried out using fresh steel surface and new portion of the AGS solution. All CPP and EIS experiments were carried out after 1 h and 24 h immersion in the AGS electrolyte.

## 4. Conclusions

The corrosion of cast iron in freely aerated stagnant AGS electrolyte at room temperature using gravimetric and electrochemical measurements after varied exposure periods of time was reported. The results obtained by WL indicated that increasing the immersion time from 2 days to 10 days increases the weight-loss. Electrochemical (CPP, OCP, and EIS) measurements taken after 1 h and 24 h showed that the increase of immersion time decreases the corrosion of cast iron through decreasing its anodic, cathodic, and corrosion currents and corrosion rate, while increasing the polarization and solution resistances. The SEM image taken for the surface of the cast iron coupon that was immersed for 10 days in AGS showed that the presence of a thick layer of corrosion products fully covers the surface. The EDX profile analysis obtained for the cast iron surface after 10 days confirmed that the presence of high amount of oxygen and low amounts of iron compared to its content in the original coupon, which indicates that the corrosion product layer mainly consists of iron oxides. These results are consistent and confirm that the increase of immersion time decreases the corrosion of cast iron in AGS due to the formation of a thick layer of iron oxides that covers the surface and decreases its dissolution.
